# Case Report: An Internal Mammary Rhabdomyosarcoma After Mastectomy and Systemic and Radiation Therapy in a Patient With Breast Cancer

**DOI:** 10.3389/fonc.2021.751758

**Published:** 2021-10-26

**Authors:** Dan-Qiong Wang, Jing-Yi Zhang, Jing Li, Jian-Ming Ying, Xiang Wang, Ying Fan, Shu-Lian Wang

**Affiliations:** ^1^ Department of Radiation Oncology, National Cancer Center/National Clinical Research Center for Cancer/Cancer Hospital, Chinese Academy of Medical Sciences and Peking Union Medical College, Beijing, China; ^2^ Department of Medical Oncology, National Cancer Center/National Clinical Research Center for Cancer/Cancer Hospital, Chinese Academy of Medical Sciences and Peking Union Medical College, Beijing, China; ^3^ Department of Diagnostic Radiology, National Cancer Center/National Clinical Research Center for Cancer/Cancer Hospital, Chinese Academy of Medical Sciences and Peking Union Medical College, Beijing, China; ^4^ Department of Pathology, National Cancer Center/National Clinical Research Center for Cancer/Cancer Hospital, Chinese Academy of Medical Sciences and Peking Union Medical College, Beijing, China; ^5^ Department of Breast Surgery, National Cancer Center/National Clinical Research Center for Cancer/Cancer Hospital, Chinese Academy of Medical Sciences and Peking Union Medical College, Beijing, China

**Keywords:** post-radiation soft tissue sarcomas, rhabdomyosarcomas, breast cancer, radiation therapy, case report

## Abstract

Post-radiation soft tissue sarcomas (PRSTSs) are rare secondary malignancies. In this report, we describe the clinical presentation of a 52-year-old woman who underwent postmastectomy radiation therapy (PMRT) for left-sided breast cancer 2.7 years ago and presented with a left internal mammary mass and left interpectoral nodule on computed tomography. On further evaluation, she was diagnosed with internal mammary rhabdomyosarcoma and interpectoral nodal breast cancer relapse, and was treated with chemotherapy, followed by surgery and endocrine therapy. She developed left pleural metastases and is currently receiving targeted therapy. Internal mammary rhabdomyosarcomas are rare among PRSTSs and pose a diagnostic challenge for patients with breast cancer. Histological evaluation is important for the differential diagnosis of breast cancer relapses with secondary malignancies. The management of post-radiation thoracic rhabdomyosarcomas is challenging, and the prognosis is poor.

## Introduction

Post-radiation soft tissue sarcomas (PRSTSs) occur in <1% of post-radiation patients. The most common site of PRSTSs is the thoracic region, which is frequently irradiated due to breast cancer or Hodgkin’s lymphoma (1). Breast cancer is known to be the most frequent original cancer of PRSTSs (1–3). The 15-year cumulative incidence of PRSTSs for breast cancer is 0.28% (4). Although PRSTSs of breast cancer are generally rare, they are increasing in number given the long-term survival of patients receiving radiation therapy (RT). Meanwhile, the use of systemic therapy may also contribute to an increase in the risk of PRSTSs (5). Therefore, clinicians should be aware of the differential diagnosis between PRSTSs and locoregional relapse.

In 1948, Cahan et al. established some criteria to define the essential characteristics of radiation-induced sarcomas of the bone (6), namely: i) the tumor must arise within, or adjacent to, a previously irradiated field; ii) the tumor should arise at least 6 months after the cessation of RT; and iii) histological confirmation of a sarcoma, distinct from the patient’s prior malignancy. These criteria have been modified over time, especially the duration of latency and the inclusion of soft-tissue sarcomas. Here, we report a rare case of internal mammary rhabdomyosarcoma and interpectoral nodal breast cancer relapse in a 52-year-old woman 2.7 years after postmastectomy radiation therapy (PMRT) and systemic therapy for breast cancer. The article was written according to the CAse REport (CARE) guidelines, and the CARE checklist is provided in **Supplementary Table 1**.

## Case Description

The patient was a 52-year-old healthy woman who was originally diagnosed with left-sided breast cancer in May 2017 (then age, 48 years). She underwent mastectomy and axillary lymph node dissection. Pathology indicated grade II, non-specific, invasive carcinoma, with a tumor size measuring 1.2×1.0 cm, and the presence of lymphovascular invasion. Immunohistochemistry (IHC) showed that the tumor was positive for estrogen receptor (ER) (80%), progesterone receptor (PR) (70%), Ki-67 (25%), and negative for HER2 (1+). Two out of 23 removed lymph nodes showed metastases, with extracapsular extension. The patient’s disease was staged as pT1cN1aM0.

From June to October 2017, she received six cycles of chemotherapy (epirubicin 60 mg d1, 2 + paclitaxel liposome 270 mg d3, q21d). Because of the grade 4 agranulocytosis with fever after the first cycle, the regimens were adjusted to epirubicin 60 mg d1, 50 mg d2 + paclitaxel liposome 240 mg d3 for the remaining five cycles.

In November 2017, she received PMRT, which consisted of 43.5 Gy in 15 daily fractions to the chest wall and supra/infraclavicular fossa, but did not intend to include the internal mammary nodal region. The chest wall was irradiated with 6 MeV electron, while the supra/infraclavicular fossa was irradiated with 6 MV x-rays by using three-dimensional conformal radiation therapy (3D-CRT) technique. Then, she was prescribed oral tamoxifen and underwent regular follow-up.

**Figure 1:**
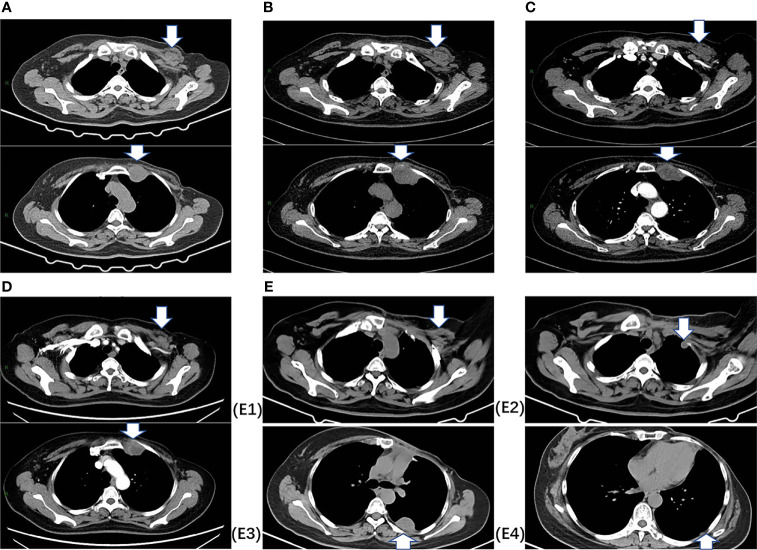
Chest CT images. **(A)** At baseline (July 2020), CT indicated a 2.7 cm×1.7 cm nodule in the left interpectoral region, and a 3.5 cm×3.0 cm mass in the left internal mammary area. **(B)** After two cycles of chemotherapy (September 2020), CT indicated stable disease (SD) with a decreased nodule size (2.4×1.5 cm) and a slight increase in the mass size (4.5×3.3 cm). **(C)** After four cycles of chemotherapy (November 2020), CT indicated SD with a decreased nodule size (2.2×1.2 cm) and mass (4.4×3.1 cm). **(D)** After six cycles of chemotherapy (February 2021), CT indicated SD with a decreased nodule size (1.5×0.7 cm) and mass (3.6×3.1 cm). **(E)** After surgery, **E1** shows the left interpectoral node shrinkage to 1.2×0.8 cm in size, and **E2, E3, E4** show appearance of multiple left pleural metastatic nodules.

In July 2020, 2.7 years after the PMRT, chest computed tomography (CT) for routine examination revealed a 2.7 cm×1.7 cm nodule in the left interpectoral region, and a 3.5 cm×3.0 cm mass in the left internal mammary area (**Figure 1**). She had no symptoms and there were no palpated nodules on physical examination. Ultrasound-guided 22-gauge needle biopsy of the interpectoral nodule was performed and histological examination revealed carcinoma cells. On IHC, the tumor was positive for AE1/AE3 and ER (10%). To further elucidate the molecular subtype of the recurrence, ultrasound-guided 16-gauge needle biopsy of the left internal mammary mass was performed, which showed few malignant tumor cells on hematoxylin-eosin (HE) staining. IHC showed that the tumor was positive for vimentin, desmin, myogenic differentiation 1 (myoD1), and Ki-67 (70%), and negative for S-100, smooth muscle actin (SMA), cytokeratin 18 (CK18), AE1/AE3, ER, and PR, suggesting spindle cell rhabdomyosarcoma. Then, ultrasound-guided 16-gauge needle biopsy of the interpectoral nodule was performed, and histology suggested grade II, non-specific, invasive carcinoma, and IHC showed that the tumor was positive for ER (70%), PR (20%), Ki-67 (70%), and androgen receptor (AR) (60%) and negative for HER2, cytokeratin 5/6 (CK5/6), EGFR, vimentin, myoD1, and desmin, confirming breast cancer recurrence. Bone-emission CT showed slightly increased intake of the medial anterior segment of the left second rib, which was considered as tumor erosion. Next-generation sequencing (NGS) revealed that she did not carry germline mutations in *BRCA1, BRCA2, MLH1*, and *MSH2* genes. After staging workup with abdominal ultrasonography, she was diagnosed with post-radiation rhabdomyosarcoma cT1N0M0Gx, stage IA, and interpectoral nodal recurrence from breast cancer.

**Figure 2:**
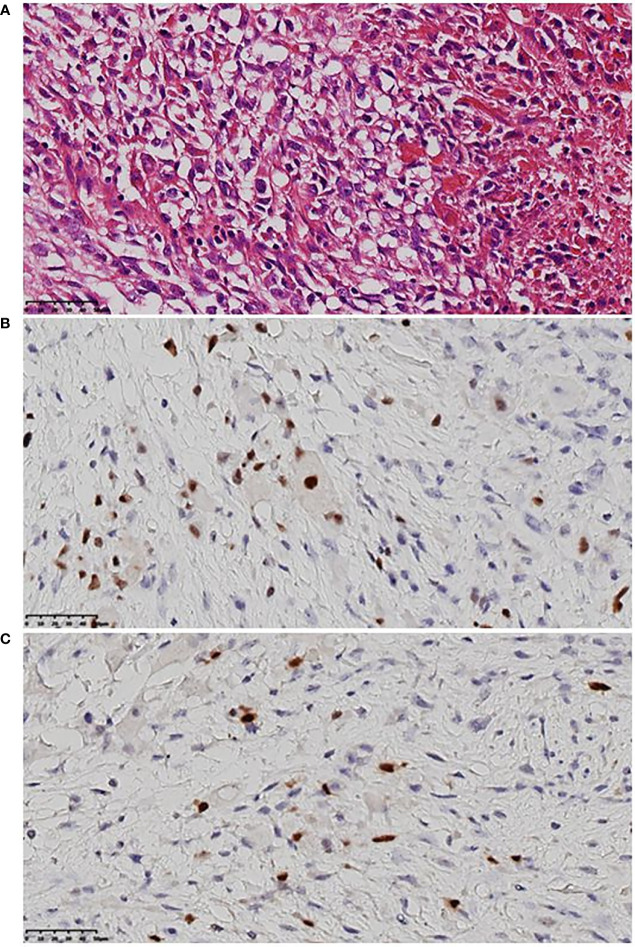
Representative histologic images of rhabdomyosarcoma. **(A)** Malignant mesenchymal tumor, mainly spindle cells, clear cytoplasm or acidophilic tumor cells, severe atypia, large and hyperchromatic nuclei, irregular karyotype, and visible mitotic image. **(B)** MyoD1 (+) by immunohistochemistry. **(C)** Myogenin (focal +) by immunohistochemistry.

**Figure 3:**
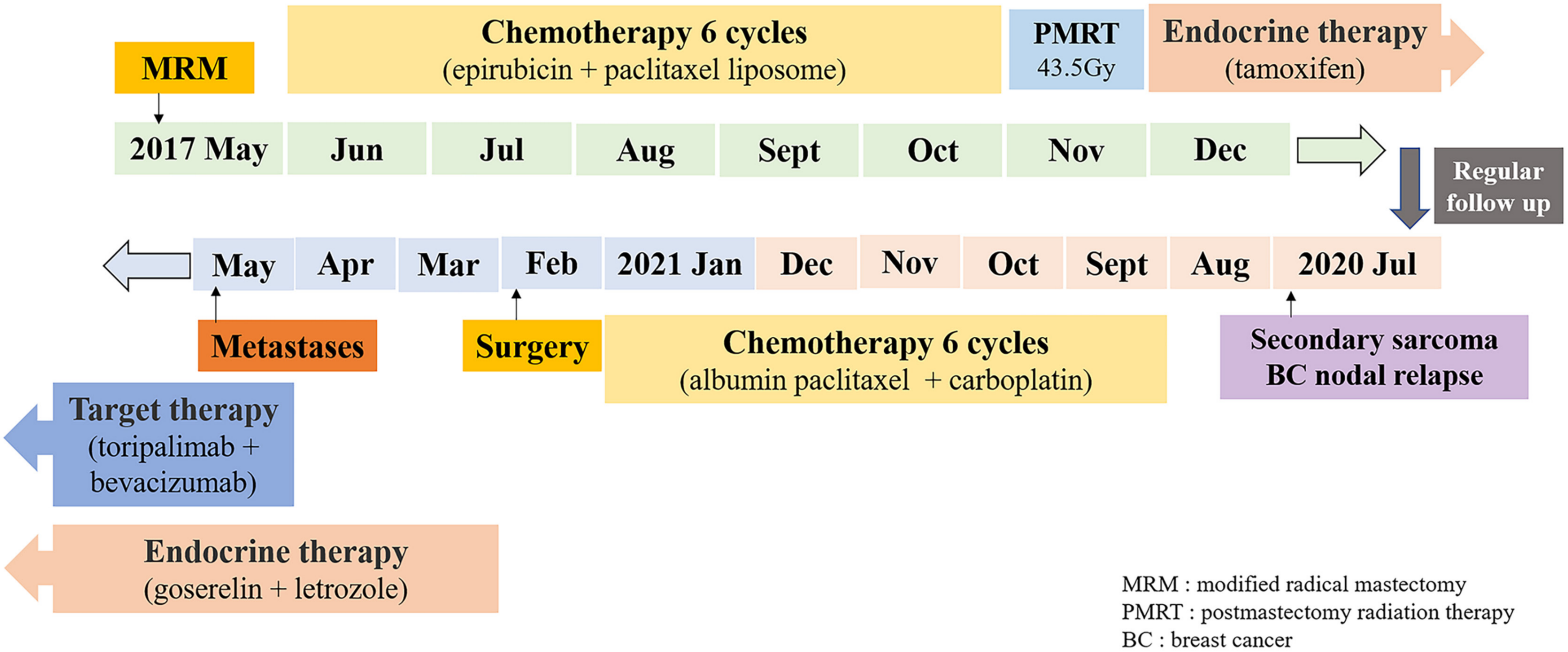
Timeline of historical and current treatments.

**Figure 4:**
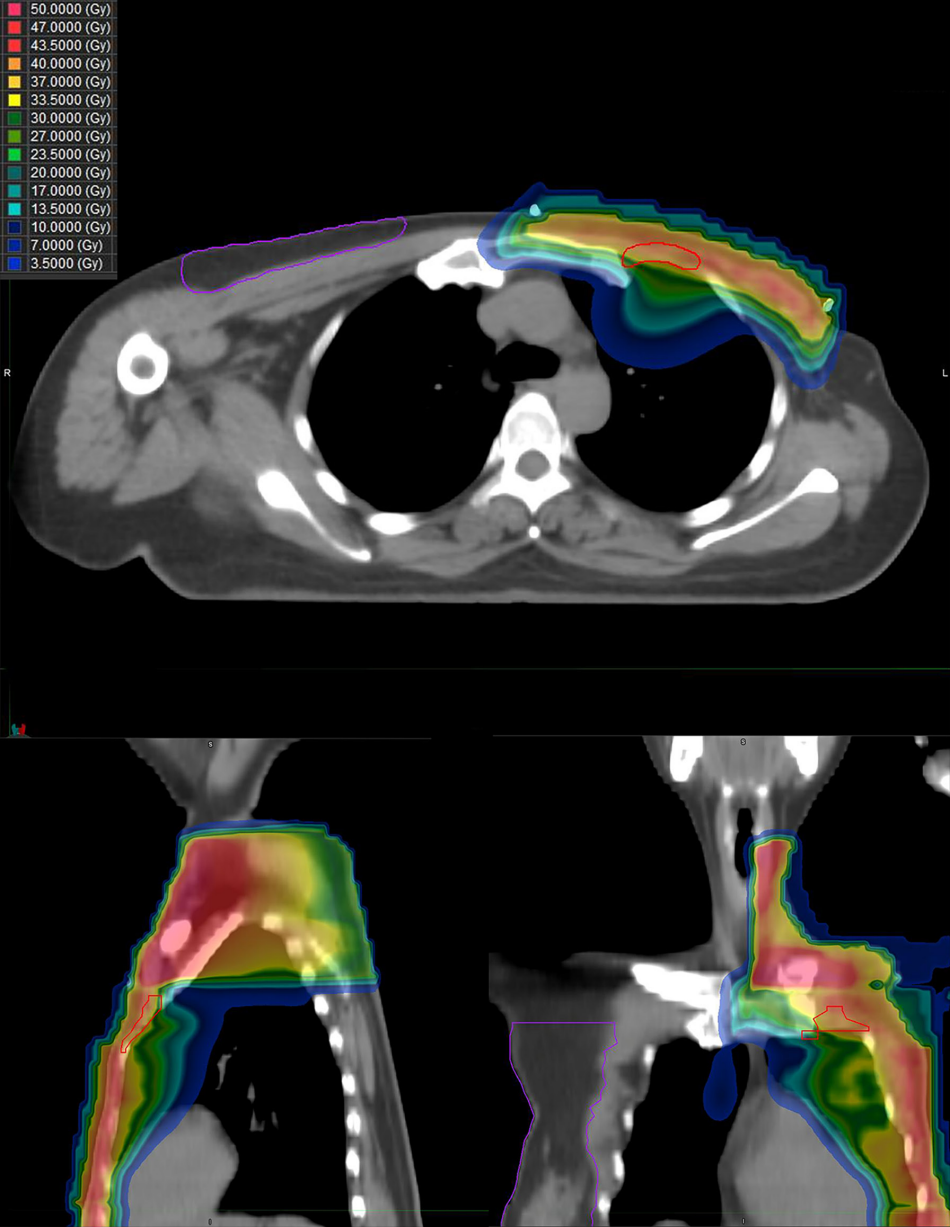
The dose distribution of PMRT the patient received for breast cancer. Upper image, horizontal view; left image, sagittal view; and right image, coronal view.

From September 2020 to January 2021, she received six cycles of chemotherapy, consisting of albumin paclitaxel 40 mg and carboplatin 700 mg, q21d. She tolerated the treatment well, developing grade I anemia, grade II vomiting, and grade II hyperlipidemia after chemotherapy. CT evaluation showed stable disease (SD) after 2, 4, and 6 cycles (**Figure 1**). After multidisciplinary team discussion in February 2021, she underwent a wide local resection of internal mammary mass along with adjacent ribs, sternum, and left upper lung. After the tumor was completely resected with 4-cm margins, intraoperative frozen section confirmed a negative margin. Macroscopically, the resected tumor measured 3 cm in diameter, and involved the sternum, rib, and left upper lung. Microscopically, the lesion consisted of spindle malignant cells, and IHC revealed that the tumor was positive for desmin (focal +), myoD1 (+), myogenin (focal +), Ki-67 (40%), and CK (focal +), and negative for CK7, SMA, ER, and PR, indicating rhabdomyosarcoma (**Figure 2**). After a normal recovery from surgery, she received goserelin and letrozole from March 2021. In May 2021, her chest CT showed that the left interpectoral node shrank to 1.2×0.8 cm in size; however, multiple left pleural metastatic nodules appeared (**Figure 1**). She refused any further chemotherapy because of fear of vomiting and disappointment with progression on chemotherapy. A complementary IHC revealed that the internal mammary tumor was positive for programmed cell death ligand 1 (PD-L1) (22C3): combined positive score (CPS) 10. From May 2021, she received programmed cell death protein 1 (PD1) antibody toripalimab 240 mg d1 and bevacizumab 400 mg d1, q21d, and has meanwhile continued her endocrine therapy. The timeline of her historical and current treatments is illustrated in **Figure 3**. Her PMRT treatment plan was retrieved, and the unintentional mean radiation dose to the left internal mammary region, where the rhabdomyosarcoma originated, was estimated as 32.5 Gy in 15 fractions (**Figure 4**).

## Discussion

Our case report is significant, because we believe it is extremely rare for a patient to develop both breast cancer recurrence and PRSTSs in two different lymphatic drainage regions that had been exposed to radiation 2.7 years ago. The post-radiation rhabdomyosarcoma was an accidental finding, as the specimen from the first biopsy of the interpectoral node was not adequate for elucidating the molecular subtype and hence, a second biopsy of the internal mammary mass was performed. Routinely, it is not required to perform histological analysis for all recurrent lesions. However, the present case suggests that because all lesions occurred in the previous radiation field, histological confirmation could be useful because of the possible development of PRSTSs.

Radiation therapy is associated with an increased risk of developing a secondary in-field PRSTSs (HR, 4.1), especially angiosarcomas (HR, 8.97), in breast cancer patients within a median latent period of 7 years. The risk for development of PRSTSs (at all sites) increased in the third year after diagnosis of breast cancer, peaked at 8–12 years, and then decreased again (7). Similarly, it has been reported that the median time from the diagnosis of original cancer to PRSTSs was 11 years (1–3). As for the present case, thoracic rhabdomyosarcoma is rare not only as PRSTSs but also as sporadic sarcoma (8). The most common histological type of PRSTSs was undifferentiated pleomorphic sarcoma, followed by angiosarcoma (2), whereas sporadic rhabdomyosarcoma occurred frequently in the head and neck region and the genitourinary tract. There is a relationship between the dose of radiation received by soft tissue sarcoma sites during breast cancer RT and the risk of developing these secondary sarcomas. Hang et al. found that the risk of angiosarcomas was 15.9-fold higher and the risk of other soft tissue sarcomas was 2.2-fold higher among women treated by RT than among those who were not (9). Rubino et al. reported that among women treated with RT, those who received >14 Gy had a higher risk of sarcoma than those who received <14 Gy, and the odds-ratios (ORs) were 1.6 and 30.6 for women who received 14–44 Gy and at least 45 Gy at the site of the sarcoma, respectively (4). Henderson et al. reported that anthracycline exposure was associated with sarcoma risk (OR=3.5) in childhood cancer survivors, after adjusting for radiation dose, other chemotherapy, and primary cancers (5). In addition, genetic susceptibility was associated with increased risk of malignancies. There was a significantly higher prevalence of post-radiation sarcoma in patients with breast cancer and Li-Fraumeni syndrome (LFS) than non-LFS breast cancer patients (6% vs. 0.03%, p<0.001) (10). Our patient was not *BRCA1, BRCA2, MLH1*, and *MSH2* germline mutations carrier but had received anthracycline-based chemotherapy and 32.5 Gy of radiation in left internal mammary site, therefore her rhabdomyosarcoma is likely attributed to previous RT and chemotherapy.

PRSTSs has worse outcomes than sporadic sarcomas (2, 11). Patients with PRSTSs had a 5-year OS of 38%–68% (2, 7). Surgery with curative intent is the mainstay of therapy. Negative surgical margins are usually associated with better OS (1, 2, 12). The management of the present case is rather complicated and both secondary rhabdomyosarcoma and breast cancer recurrence were taken into consideration. Our patient received chemotherapy followed by curative surgery. Unfortunately, she developed multi-pleural metastases shortly after surgery. Patients with thoracic rhabdomyosarcoma have a poorer prognosis than those with rhabdomyosarcoma at other sites, which might be due to the higher percentage of advanced-stage tumors and technical difficulties of local therapy at the thoracic site. The rhabdomyosarcoma had involved the left upper lung and pleura in the present case. Tumors may extend or disseminate along pleural surfaces and be deceiving in their size and boundaries; hence, more vigorous measures to ensure local and distant control may be needed. Based on the evidence that PD-1 blockade combined with vascular endothelial growth factor (VEGF) inhibitor showed acceptable toxic effects and preliminary activity in advanced sarcomas (13), our patient was willing to try PD1 antibody and bevacizumab, but further follow-up is warranted.

The present study indicates the importance of differential diagnosis between tumor relapse and PRSTSs for a mass developed in the lymph drainage site for breast cancer patients with a history of RT. We highlight this case with the intention of alerting clinicians to obtain histological confirmation for any in-field relapse.

## Conclusion

Internal mammary rhabdomyosarcomas are extremely rare among PRSTSs and pose a diagnostic challenge for patients with breast cancer. Histological evaluation is important for differential diagnosis of breast cancer relapses with secondary malignancies.

## Data Availability Statement

The original contributions presented in the study are included in the article/**Supplementary Material**. Further inquiries can be directed to the corresponding authors.

## Ethics Statement

Written informed consent was obtained from the individual(s) for the publication of any potentially identifiable images or data included in this article.

## Author Contributions

D-QW, S-LW, and YF performed image acquisition and completed the manuscript. All authors contributed to the article and approved the submitted version.

## Funding

This work was supported by the CAMS Innovation Fund for Medical Sciences (2020-I2M-C&T-B-075).

## Conflict of Interest

The authors declare that the research was conducted in the absence of any commercial or financial relationships that could be construed as a potential conflict of interest.

## Publisher’s Note

All claims expressed in this article are solely those of the authors and do not necessarily represent those of their affiliated organizations, or those of the publisher, the editors and the reviewers. Any product that may be evaluated in this article, or claim that may be made by its manufacturer, is not guaranteed or endorsed by the publisher.
