# Supplementary material for: Case Report: An Internal Mammary Rhabdomyosarcoma After Mastectomy and Systemic and Radiation Therapy in a Patient With Breast Cancer
Source: Front Oncol. 2021 Oct 26;11:751758. doi: 10.3389/fonc.2021.751758 (PMC8576335; doi:10.3389/fonc.2021.751758)
Supplement: Supplementary file 1 [file Table_1.pdf]

CARE case report guidelines

CARE Checklist of information to include when writing a case report

(cc) BY-NC-ND

Topic	Item	Checklist item description	Reported on Line
**Title**	1	The diagnosis or intervention of primary focus followed by the words "case report"	0
**Key Words**	2	2 to 5 key words that identify diagnoses or interventions in this case report, including "case report"	22-23
**Abstract (no references)**	3a	Introduction: What is unique about this case and what does it add to the scientific literature?	31-33
	3b	Main symptoms and/or important clinical findings	25-28
	3c	The main diagnoses, therapeutic interventions, and outcomes	28-31
	3d	Conclusion—What is the main "take-away" lesson(s) from this case?	32-34
**Introduction**	4	One or two paragraphs summarizing why this case is unique (**may include references**)	35-51
**Patient Information**	5a	De-identified patient specific information.	53-54
	5b	Primary concerns and symptoms of the patient.	53
	5c	Medical, family, and psycho-social history including relevant genetic information	79-81
	5d	Relevant past interventions with outcomes	54-67
**Clinical Findings**	6	Describe significant physical examination (PE) and important clinical findings.	68-70
**Timeline**	7	Historical and current information from this episode of care organized as a timeline	102-103;167
**Diagnostic Assessment**	8a	Diagnostic testing (such as PE, laboratory testing, imaging, surveys).	68-83
	8b	Diagnostic challenges (such as access to testing, financial, or cultural)	109-113
	8c	Diagnosis (including other diagnoses considered)	83-85
	8d	Prognosis (such as staging in oncology) where applicable	84
**Therapeutic Intervention**	9a	Types of therapeutic intervention (such as pharmacologic, surgical, preventive, self-care)	86-95
	9b	Administration of therapeutic intervention (such as dosage, strength, duration)	86-87
	9c	Changes in therapeutic intervention (with rationale)	145-147
**Follow-up and Outcomes**	10a	Clinician and patient-assessed outcomes (if available)	100-102
	10b	Important follow-up diagnostic and other test results	96-98
	10c	Intervention adherence and tolerability (How was this assessed?)	98-99
	10d	Adverse and unanticipated events	97
**Discussion**	11a	A scientific discussion of the strengths AND limitations associated with this case report	107-113
	11b	Discussion of the relevant medical literature **with references**.	106-150
	11c	The scientific rationale for any conclusions (including assessment of possible causes)	114-134
	11d	The primary "take-away" lessons of this case report (without references) in a one paragraph conclusion	148-150
**Patient Perspective**	12	The patient should share their perspective in one to two paragraphs on the treatment(s) they received	87;98-99
**Informed Consent**	13	Did the patient give informed consent? Please provide if requested	Yes ☑ No ☐
